# Effects of balance training with visual input manipulations on balance performance and sensory integration in healthy young adults: a randomized controlled trial

**DOI:** 10.1038/s41598-024-79736-x

**Published:** 2024-11-19

**Authors:** Jakob Ketterer, Albert Gollhofer, Steffen Ringhof, Lorenz Assländer, Urs Granacher, Dominic Gehring

**Affiliations:** 1 Department of Sport and Sport Science, Exercise and Human Movement Science, University of Freiburg, Freiburg, Germany; 2 Department of Diagnostic and Interventional Radiology, Faculty of Medicine, University Medical Center Freiburg, University of Freiburg, Freiburg, Germany; 3 Department of Sport Science, Human Performance Research Centre, University of Konstanz, Konstanz, Germany; 4 Department of Sport and Sport Science Exercise and Human Movement Science, University of Freiburg, Sandfangweg 4, 79102 Freiburg, Germany

**Keywords:** Virtual reality, Sensory reweighting, Sensory-to-motor transformation, Feedback loop, Balance control model, Randomized controlled trial, Motor control, Sensory processing, Rehabilitation

## Abstract

Although balance training can improve balance across various populations, the underlying mechanisms, such as how balance training may alter sensory integration, remain unclear. This study examined the effects of balance training with visual input manipulations provided by virtual reality versus conventional balance training on measures of postural sway and sensory integration during balance control. Twenty-two healthy young adults were randomly allocated into a balance training group (BT) or a balance training with virtual reality group (BT + VR). The BT received traditional balance training, while the BT + VR additionally received visual manipulations during the 4-week balance training to elicit sensory conflicts. Static balance was measured in the form of center of pressure (COP) sway speed in trained (eyes open) and untrained (eyes closed) balance conditions. A model-based analysis quantified the sensory integration and feedback characteristics of the balance control mechanism. Herein, the visual weight quantifies the contribution of visual orientation information to balance while the proportional and derivative feedback loop-gains correct for deviations from the desired angular position and angular velocity, respectively. Significant main time effects were observed for the visual sensory contribution to balance (*p* = 0.002, $\:{\eta\:}_{p}^{2}$ = 0.41) and for the derivative feedback loop-gain (*p* = 0.011, $\:{\eta\:}_{p}^{2}$ = 0.29). Significant group-by-time interactions were observed for COP sway speed in the untrained task (*p* = 0.023, $\:{\eta\:}_{p}^{2}$ = 0.23) in favor of BT + VR and in the proportional feedback loop-gain, with reductions only in the BT + VR group (*p* = 0.043, $\:{\eta\:}_{p}^{2}$ = 0.2). BT + VR resulted in larger performance improvements compared with traditional BT in untrained tasks, most likely due to reduced reliance on visual information. This suggests that the systematic modulation of sensory inputs leads to enhanced capacity for motor adaptation in balance training.

**Supplementary Information:**

The online version contains supplementary material available at 10.1038/s41598-024-79736-x.

## Introduction

Balance training has the potential to improve balance control in different populations across the lifespan^1–3^. There is evidence in the scientific literature showing that balance training is effective in (i) enhancing measures of physical fitness and sport-specific performance^4,5^, (ii) preventing injuries in athletes^6,7^, (iii) reducing the risk of falls in older adults^8,9^, and (iv) accelerating rehabilitation and return-to-sport processes^10^. To control balance in everyday and sports-related situations, sensory information about the body’s orientation in space is an immediate prerequisite. Specifically, the central nervous system (CNS) integrates information from visual, vestibular, and somatosensory systems and generates the required stabilizing joint torques based on the sensory-derived estimate of body orientation^11^. When standing on a firm surface, ankle joint moments change the position of the center of pressure (COP) which impacts on the center of mass (COM) accelerations^12^. The resultant change in body orientation is detected by the sensory systems, thus providing consistent and reliable feedback.

Balance training, in contrast, typically involves static and dynamic balance tasks on unstable surfaces (e.g., balance pads) which reduces the reliability of the afferent input from the somatosensory system in encoding body orientation in space. Thus, it provides different sensory integration for balance control compared with stable surface conditions. More specifically, unstable surfaces impact the relation of ankle joint moments and COP displacement by deforming the support surface, causing increased COP displacements and COM accelerations^12^. Ultimately, changes in the muscle length in the lower extremities no longer match changes in body orientation^13^. In essence, balance exercises on unstable surfaces intentionally create situations where the CNS must adapt to different balance threatening situations which challenge sensory integration beyond balance control on stable ground^13,14^. In this context, sensory reweighting is an important mechanism that allows the CNS to adapt to balance-threatening situations by prioritizing more reliable sensory information^15^. For example, postural perturbations through surface manipulations have been associated with upweighting of visual and vestibular information relative to somatosensory information^16–18^. Consequently, the relevance of a sensory system may vary depending on its importance in a specific balance task. For instance, judo players and well-trained gymnasts, compared to untrained participants, exhibit less disruption in balance control when closing their eyes, suggesting a reduced reliance on visual information^19,20^. In contrast, dancers do not show this pattern, indicating a greater dependency on visual information in their training routines^19^. However, these research findings come from cross-sectional studies, which do not allow to deduce cause-and-effect relationships in (visual) sensory integration as a result of balance training. In addition, findings of these studies are based on overall postural sway measures, which provide valuable information about the state of the balance system, but are constrained in discriminating changes in the sensory integration mechanism from other balance control processes that affect postural sway^21^. Hence, whether balance training leads to changes in sensory integration remains elusive. The current body of research lacks comprehensive insights from longitudinal studies combined with appropriate methodological approaches to quantify the modifications in sensory integration attributable to balance training.

This study aims to bridge this gap by employing system-identification methods from control theory to quantitatively model the sensory integration mechanism for balance control before and after balance training^11,22^. Further, we apply traditional balance training in combination with targeted visual sensory manipulations using virtual reality (VR) to leverage the sensory reweighting mechanism for balance training by manipulating more than one sensory modality^23^. Unlike conventional balance training types that typically offer binary visual inputs (eyes open or closed), VR allows to induce sensory conflicts, decoupling the visual from vestibular and somatosensory feedback^24^. The additional demands on sensory integration and balance control might lead to greater training-induced improvements in balance performance^25^. Our previous research identified visual input manipulations within VR that induce persistent balance perturbations^26,27^. Here, we use our previous findings and apply them in the context of a balance training study.

Therefore, this study aimed to investigate the effects of balance training augmented with visual sensory manipulations through VR on balance performance and sensory integration in healthy young adults. Referring to Lee et al.^28^, who reported greater balance improvements and reduced reliance on visual information following balance training with visual disruption in patients with chronic ankle instability, we hypothesized that participants undergoing balance training with VR exhibit greater improvements in balance performance compared to those undergoing traditional balance training without VR. Furthermore, we expected that VR-related balance training would alter the relative contribution of sensory inputs for balance control with a reduced reliance on visual information^28,29^.

## Methods

### Participants

We performed an a priori power analysis (G*Power 3.1), utilizing effect sizes derived from a prior study investigating the effects of a similar balance training program on steady-state balance performance (COP sway speed in single-leg stance) in healthy young adults^30^. Results revealed that a total of *N* = 20 participants would be needed to detect the observed effect size using the input parameters $\:f$ = 0.35, an alpha error = 0.05 and a power of 95%. Sample size determination referred to the calculation of a mixed ANOVA, focussing on the within-between interaction across two time points. Consequently, a total of *N* = 22 male and female participants, aged 18 to 40 years, were enrolled. Participants were only eligible if they were healthy without any present or past injury or illness that would affect participation in balance tests or training. Participation was not possible if individuals practiced systematic balance training prior to the start of the study. Before study inclusion, participants were informed about the study procedures, risks and benefits related to the study and provided written informed consent. The study was conducted at the University of Freiburg, Germany, received ethical clearance from the local ethics committee and the study procedures were in accordance with the latest version of the declaration of Helsinki.

### Experimental setup

During testing, participants stood in upright position on a force-platform (AMTI BP600900, Watertown, MA, United States), which measured the position of the COP in the lateral and anterior-posterior directions at a sampling frequency of 1,000 Hz. During testing conditions that required manipulated visual input, participants’ visual surround was provided through a VR system using a head-mounted display (HTC Vive pro eye, HTC Corporation, Taoyuan City, Taiwan) with a spatial resolution of 1440 × 1600 pixels per eye, a temporal resolution of 90 Hz, and a field of view of 110°. Graphics were rendered using an AMD Ryzen 9 3900X processor and Nvidia GeForce RTX 2080 graphics card. The HTC Vive system incorporated a lighthouse tracking system to monitor head position and orientation, dynamically updating the VR perspective accordingly. Within the VR, participants were immersed in a synthetic replication of our laboratory^27^. The VR space was programmed and controlled using a custom-written C#-code in Unity3d (Unity Technologies, San Francisco, CA). To create sensory conflicts and induce balance perturbations in the according experimental condition and the training protocol, the visual scene was tilted in the frontal plane, simulating a roll of the virtual environment around a virtual axis positioned between the ankle joints. This axis mirrored the rotation experienced by the external world on the retina during medio-lateral body sway.

To examine the sensory integration mechanism underlying balance control, we utilized the VR application developed and validated by Assländer et al.^22^. This VR-application comprised a half-cylindric screen (radius 1 m) with vertical and horizontal stripes to induce systematic perturbations on the visual sensory system. The screen moved according to predefined sequences, tilting around the ankle joint axis in the sagittal plane (axis positioned 8.8 cm above the surface). The visual stimulus was generated using a pseudo-random-ternary sequence (PRTS) alternating fixed positive, negative or zero velocity. The sequence comprised 80 states, each lasting 0.25 s, resulting in a cycle period of 20 s, and was scaled to yield a peak-to-peak amplitude of 1°. Eleven single cycles were concatenated to form a final stimulus waveform of 220 s. An illustration of the VR scene and the visual stimulus can be found by Assländer et al.^22^.

A central control unit (Vicon D-Link, Vicon Motion Systems Ltd., Oxford, United Kingdom) enabled communication between the data collection computer and the visual stimulus computer, ensuring synchronized data collection with the onset of the stimulus application.

### Procedures

A parallel-group randomized controlled trial with repeated measures was conducted to evaluate the effects of balance training with or without visual input manipulations provided by VR on balance performance across different balance tasks. Further, visual sensory integration for balance control was assessed before and after the completion of the balance exercise program.

After signing the informed consent, subjects’ mass and height were measured. Upon confirming study eligibility, participants were randomly allocated to one of two intervention groups. While group 1 performed a four-week traditional balance training (BT) without VR, group 2 performed the same exercises as group 1, but with additional visual input manipulations using VR (BT + VR). A random number generator in Matlab was used for allocation sequence generation.

All participants completed the same measurements before and after the training. First, we examined postural sway across four different balance tasks to assess balance performance, task-specific and non-specific ones. After completing the balance performance assessment, we examined the sensory integration mechanism of visual information for balance control using model-based interpretations of sway responses to virtual visual scene movements^22^. Herein, participants were in upright and bipedal stance position on a foam pad (Alphapace Balance Pad, 40 × 33 × 6 cm thick), with feet positioned shoulder-width apart and hands hanging to the side, while facing the virtual half-cylindric screen^22^. Foot position was corrected if necessary to align the ankle joints with the screen’s axis of rotation. Participants were informed about the impending screen movement and instructed to maintain an upright and comfortable stance, while consistently looking straight ahead. To minimize external auditory cues, the physical environment remained quiet, with no verbal communication permitted during the trials. To reduce potential confounding factors, all tests within one individual were conducted at consistent times of the day. Additionally, participants were instructed not to perform any strenuous activity before the balance tests on the test day.

### Balance performance

To assess balance performance, participants were tested in four distinct balance tasks: (1) single-leg stance on foam (Alphapace Balance Pad, 40 × 33 × 6 cm thick) with eyes open, (2) single-leg stance on firm surface with eyes open, (3) single-leg stance on firm surface with eyes closed, and (4) semitandem-stance on foam with visual sensory input manipulations provided by VR. The single-leg conditions were performed on the dominant leg. The definition of the dominant leg was based on self-reports^31,32^. In the semi-tandem stance condition, the dominant foot was placed before the non-dominant foot and the visual scenery was tilted in the frontal plane to perturb balance. A stimulus employing a trapezoidal position profile with an amplitude of 5° and a tilting velocity of 5 °/s was utilized for visual input manipulation (for details in stimulus generation see^26^). The direction and timing of each stimulus applied were randomized, with intervals between the sudden appearance of the stimulus varying from 1 to 3 s. Following the tilting motion, the visual scene remained at the maximum excursion for a duration of 1 s before returning to the 0° position in the subsequent frame. Four of these visual input manipulations were employed within the course of the balance task.

Participants maintained their stance for 20 s, hands to the hips, with three repetitions per condition (12 repetitions in total). The order of the conditions was randomized. Recorded data were analysed off-line using Matlab (The Mathworks, Natick, United States). COP data were filtered using a fourth order low-pass Butterworth filter with a cut-off frequency of 6 Hz, and the mean COP sway speed over the 20 s trials duration was calculated. The mean value across three trials per condition was utilized for further statistical analyses.

### Balance control model

For the analysis of the balance control mechanism, subjects were in upright, bipedal stance with feet shoulder-width apart. The COM tilt around the ankle joints in anterior-posterior direction served as the primary variable for all analyses. We used the COP data to approximate the COM displacement following the method as previously described by Peterka et al.^11^. The COM height estimate above the ankle joint was obtained following Winter^33^ using participants’ anthropometrics and mass distribution Table^33^. The COM displacement time series and the COM height above the ankle joint were then used to calculate the COM tilt angle with respect to the vertical using trigonometry. The first of the eleven pseudo-random ternary sequences and the respective COM body sway angles were discarded to avoid transient responses. Subsequently, we calculated discrete Fourier transforms of each of the last ten cycles of the stimulus and COM sway response time series. Frequency response functions (FRFs) were calculated by dividing the averaged COM spectrum by the averaged stimulus spectrum. All even harmonics of the Fourier transforms of COM sway response and stimulus were discarded prior to the FRF calculation, because the PRTS stimulus has no energy at these frequencies. We further averaged across frequency to reduce the number of frequency points at higher frequencies (for details see^11,15,22^). The FRFs served as the experimental data basis to characterize each individual’s sway behavior to the visual stimulus. The experimental FRFs were interpreted by fitting the “Independent-Channel” (IC) model proposed by Peterka^15^ to the experimental data. In the IC model, body biomechanics are represented as single-link inverted pendulum. Deviations from the desired upright body orientation are detected by visual, vestibular and proprioceptive sensory systems, which are assigned a relative sensory weight based on the reliability of the sensory information. For visual scene perturbations, the focus of the current study, the visual sensory weight (W_v_) can be estimated. W_v_ quantifies the relative contribution of visual sensory orientation information to the overall corrective torque stabilizing balance. Higher values of W_v_ indicate a greater reliance on visual information for balance control compared to lower values of W_v_. A weighted combination of sensory information generates an estimate of body orientation. Estimates of body orientation are compared to an internal reference that specifies a desired upright body position, with deviations generating a feedback error signal that drives motor corrections. This feedback error signal is processed by a neural controller (NC), which represents the sensory-to-motor transformation and generation of corrective torque at the ankle joint. The NC converts deviations from upright posture into corrective ankle torque using a control mechanism with a proportional and a derivative feedback loop-gain (PD control mechanism). These feedback loop-gains determine the strength of muscle contractions in response to deviations from the internal reference (i.e., sensory error). Specifically, the proportional feedback loop-gain K_p_ corrects for angular position errors, while the derivative feedback loop-gain K_d_ corrects for angular velocity errors. Lower values of the feedback-loop gains indicate that the NC decreases its response to the feedback error signal. Since feedback-control is not instantaneous, the feedback time delay parameter T_d_ accounts for all time delays in the neural control mechanism (central processing, neural transmission, muscle activation)^15^. Lastly, the parameter K_t_ represents the contribution from a low-pass filtered positive torque-feedback loop, proposed to explain low-frequency sway characteristics with a period > 20s^34,35^. Model parameters subject to optimization are thus, *W*_*v*_, K_p_, K_d_, K_t_, and T_d_.

Model parameters were estimated for each individual using an optimization procedure that minimized the error between experimental FRFs and simulated FRFs. Specifically, we used an optimization algorithm based on a Maximum-Likelihood estimator to fit the model to the experimentally measured FRFs, adjusting the parameters to minimize an error function between experimental data and the model prediction. Model formulation and parameter estimation was performed following the methods as described by Assländer et al.^22^.

### Balance training

The balance interventions lasted four weeks with two weekly sessions and a minimum of one day of rest between sessions. Each training session lasted approximately 15 min. The intervention programs were identical with regards to training volume. The balance training was guided by the findings of a meta-analysis by Lesinski et al.^2^, who provided dose-response relations for training period, frequency etc. on balance performance in young healthy adults. The training sessions for the participants of the BT + VR group were performed onsite in our laboratory, due to the infrastructural demands of our VR-headset. Conversely, participants of the BT group received a training plan and performed the training home-based after they received extensive instructions on how to perform the balance exercises. To track and ensure adherence to the training regimen, participants kept a training diary.

**Fig. 1:**
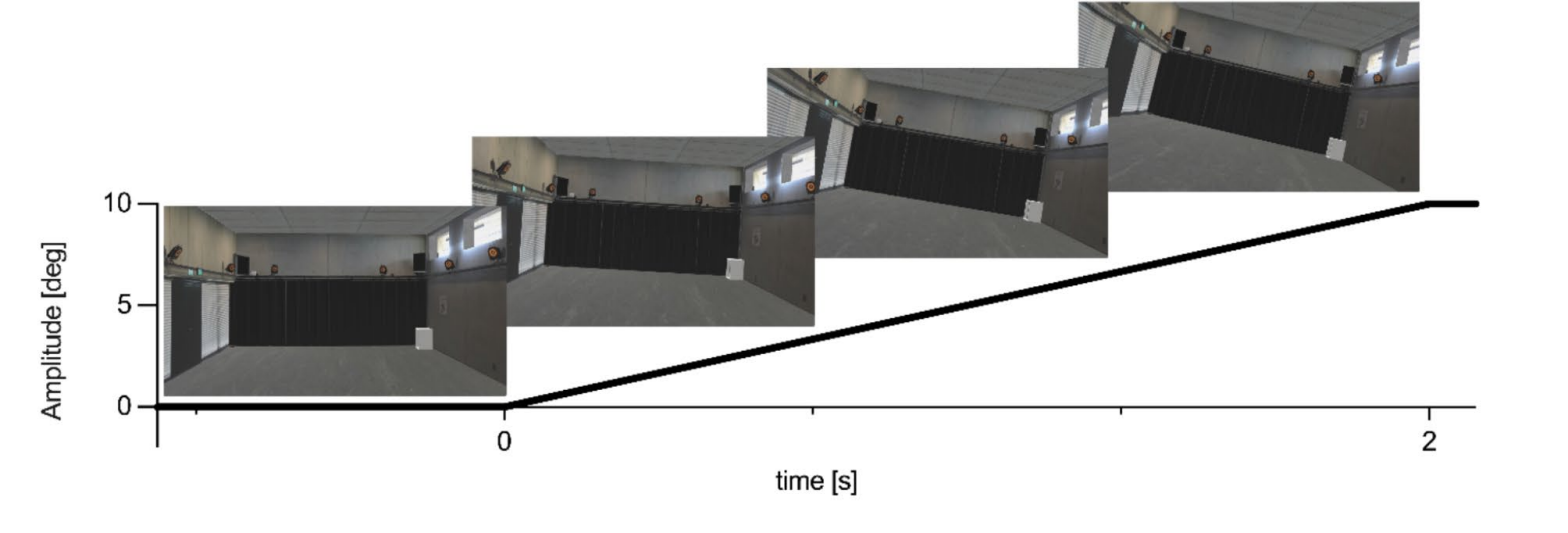
Illustration of the visual sensory manipulation applied to subjects in the BT + VR group. The images show the first-person perspective of the subject in the VR environment illustrating how the virtual scene tilts along the displayed position profile.

Both intervention groups underwent the same balance training protocol. Each training session involved ten trials of 30 s single-leg or semi-tandem stance, interspersed with 30 s resting intervals. A two-minute rest was provided after the completion of the fifth trial. With respect to the high specificity of balance training, we opted for an intervention that primarily targeted one of the tested balance tasks, namely single-leg stance on a foam pad. Therefore, all training sessions were performed in both training groups on the foam pad. For BT + VR, the virtual scenery tilted five times per balance trial, with randomized tilting direction and randomized time delays of 1 to 4 s between each tilt to introduce unpredictability into the visual input manipulations. The visual scenery tilted with a trapezoidal position profile and a tilting velocity of 5 °/s^26^ (Fig. 1). After reaching the target amplitude, the visual scene remained at the maximum excursion for a duration of 1.5 s before returning to the 0° position in the subsequent frame. The first two training sessions consisted of semi-tandem stances to familiarize the BT + VR group to the visual manipulations. This was informed by pilot measurements indicating that the participants were excessively challenged if they were trained in a one-legged stance and visual manipulations already in the first training session. To ensure consistency across the two groups, the BT group also performed the first two training sessions in semi-tandem stance. During the first training session, the visual scene tilted 10° for the BT + VR group, increasing to 20° in the second session. The third and fourth sessions introduced single-leg stances, with the visual scene tilting 5° for the BT + VR group. In training sessions six to eight, only single-leg stances were used, with the visual scene tilting 10° for the BT + VR group (see to supplementary material for details).

### Statistics

Statistical analyses were conducted using R. In a first step, assumption of variance homogeneity and normal distribution were assessed and confirmed. Normal distribution was determined by Shapiro-Wilk test and Q-Q-plot inspection. Thereafter, a mixed ANOVA was employed, incorporating the within-group factor “time” (pre vs. post) and the between-group factor “group” (BT + VR vs. BT) to examine changes in balance performance and balance control model parameters. Between-group differences in baseline parameters were evaluated using unpaired *t*-tests. In case of significant group-by-time interactions, Bonferroni adjusted and group specific *post-hoc* paired *t*-tests were computed. Percentage changes were calculated as [(post-training value – pre-training value)/pre-training value] × 100.

All data in text and tables are expressed as means ± standard deviations. The level of significance for all statistical tests was set a priori to *p* < 0.05. Effect sizes were calculated using partial eta squared ($\:{\eta\:}_{p}^{2}$) for main effects and interaction effects, with $\:{\eta\:}_{p}^{2}$ = 0.01, $\:{\eta\:}_{p}^{2}$ = 0.06, and $\:{\eta\:}_{p}^{2}$ = 0.14 indicative of small, medium, and large effect sizes, respectively. To estimate the strength of potential alterations of the investigated parameters following training, within-group effect sizes were computed using Cohen’s *d* as demarcations for small effects: 0.20 < *d* < 0.50, medium effects 0.50 < *d* < 0.80, and large effects *d* > 0.80)^36^.

## Results

**Fig. 2:**
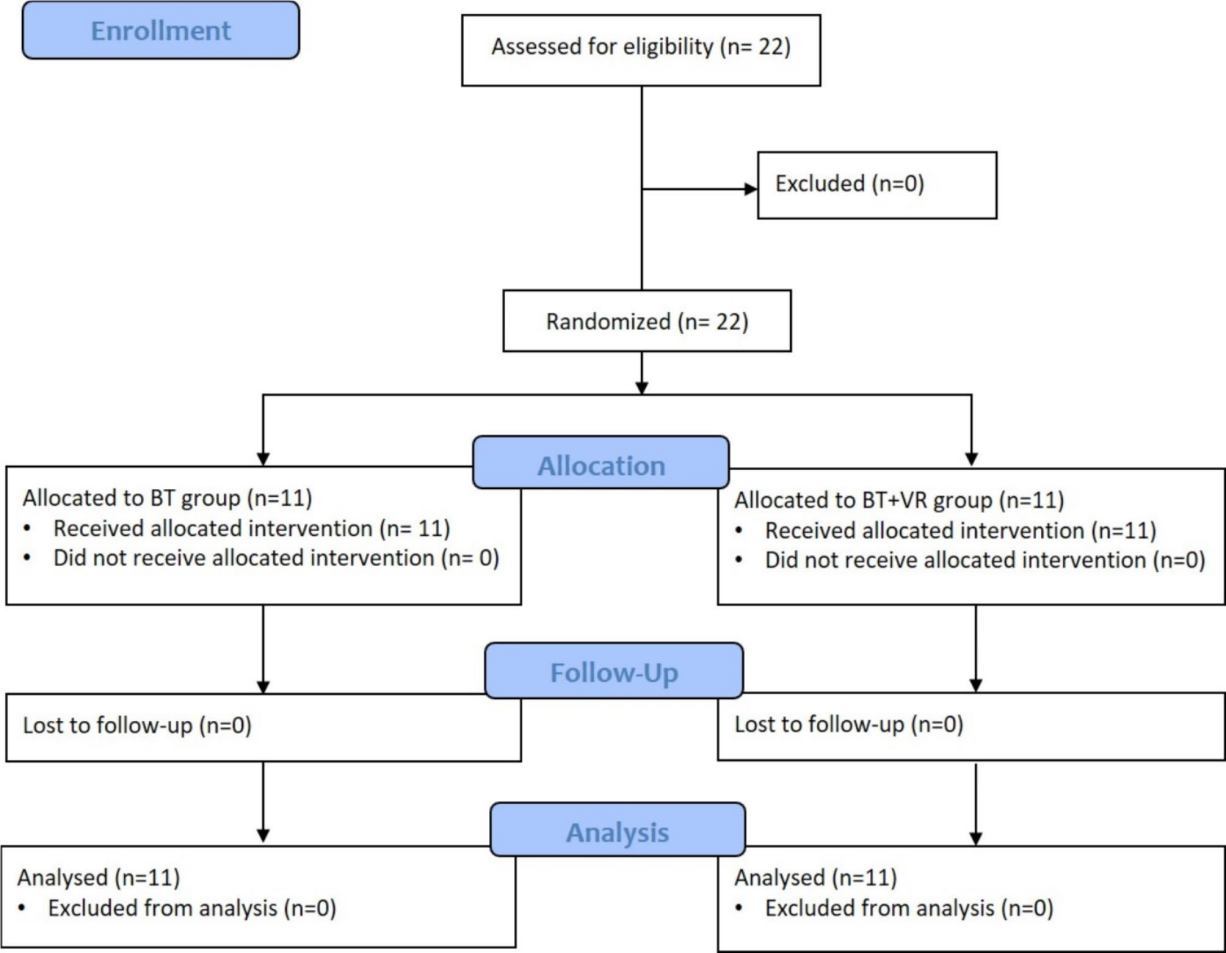
CONSORT flow diagram. BT = traditional balance training; VR + BT = balance training using virtual reality.

A total of *N* = 22 participants completed the four-week intervention period, with *n* = 11 participants in the BT group and *n* = 11 in BT + VR group (Fig. 2). All participants received the treatments as allocated. No adverse events occurred during testing and training, and all participants completed all exercise sessions, achieving 100% training adherence. No statistically significant baseline between group differences were observed for all parameters (Table 1).

**Table 1:** Baseline participant characteristics (means ± SDs).

	VR + BT (*n* = 11)	BT (*n* = 11)
Sex (m/f)	6 / 5	5 / 6
Age (yr)	22.9 ± 0.9	23.1 ± 1.1
Height (cm)	175.4 ± 9.0	174.3 ± 8.9
Mass (kg)	66.8 ± 10.1	69.4 ± 10.6
BMI (kg/m^2^)	21.6 ± 2.4	22.7 ± 1.4

BMI = body-mass-index; BT = traditional balance training; VR + BT = balance training using virtual reality.

**Table 2:** Group-specific changes in measures of balance performance and model-based parameters from pre to post.

Measure	BT + VR					BT					ANOVA *p* (η_*p*_^2^)		
	Pre		Post		∆ [%]	Pre		Post		∆ [%]	Time	Group	time × group
	M	SD	M	SD		M	SD	M	SD				
Balance Performance | COP mean sway speed													
SLS foam w/ eo [mm/s]	62.7	20.4	51.9	16.2	-17	56.6	9.3	51.6	8.3	-9	**< 0.001** **(0.58)**	0.597 (0.19)	0.069 (0.16)
SLS firm surface w/ eo [mm/s]	46.3	15.1	40.9	11.7	-12	39	6.4	40.7	5.3	4	0.321 (0.05)	0.364 (0.18)	0.065 (0.16)
SLS firm surface w/ec [mm/s]	104.2	36.7	82.8	28.8	-21	95.3	25.9	90.1	24.2	-5	**0.001** **(0.41)**	0.976 (0.00)	**0.023** **(0.23)**
STS foam w/ visual manipulations [mm/s]	78.3	27.2	44.3	12	-43	76.7	20.3	58.2	13.2	-24	**< 0.001** **(0.63)**	0.383 (0.09)	0.098 (0.13)
Model-based parameters													
W_v_ [%]	26.4	6.7	20.2	5.6	-23	26.7	5.3	23.2	3.7	-13	**0.002** **(0.41)**	0.411 (0.08)	0.339 (0.05)
K_p_ [1/deg]	1.28	0.11	1.19	0.05	-7	1.23	0.07	1.24	0.05	1	0.091 (0.14)	0.798 (0.01)	**0.043** **(0.2)**
K_d_ [s/deg]	0.48	0.05	0.46	0.04	-4	0.49	0.04	0.46	0.05	-6	**0.011** **(0.29)**	0.716 (0.04)	0.485 (0.03)
T_d_ [s]	0.19	0.027	0.196	0.02	3	0.186	0.02	0.177	0.03	-5	0.684 (0.01)	0.259 (0.30)	0.076 (0.16)
K_t_ [deg/Nm]	0.13	0.07	0.14	0.08	8	0.19	0.07	0.15	0.1	-21	0.519 (0.02)	0.236 (0.18)	0.136 (0.11)

BT = traditional balance training; VR + BT = balance training using virtual reality; COP = center of pressure; ec = eyes closed; eo = eyes open; K_**p**_& K_**d**_= proportional and derivative feedback loop-gain, normalized by mgh (subject mass*gravitational constant*center of mass height); K_**t**_= torque feedback; SLS = single-leg stance; STS = semi-tandem stance; Td = time delay; W_v_= visual sensory weight.

Significant values are in bold.

### Balance performance

**Fig. 3:**
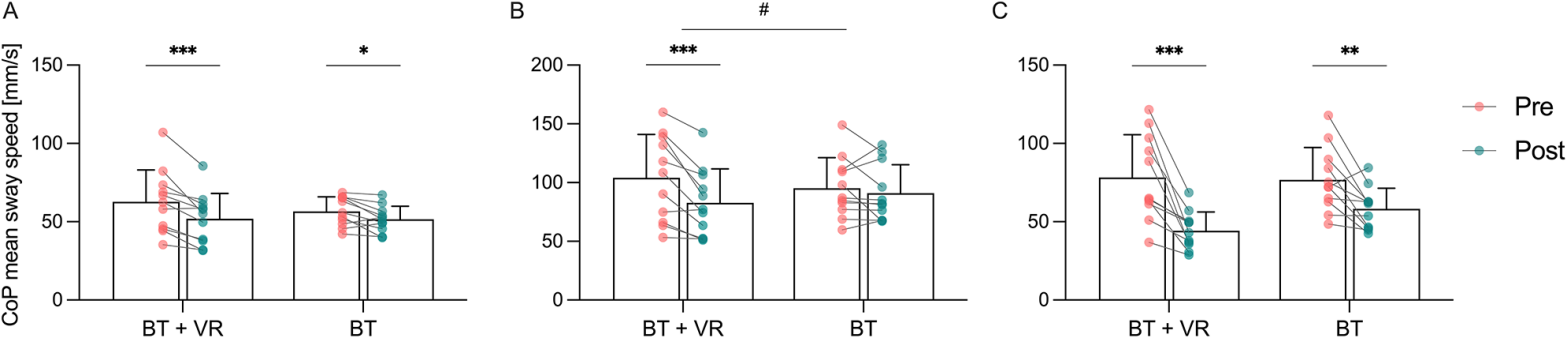
Centre of pressure (COP) mean sway speed from pre- (red) to post- (green) exercise in the balance training with virtual reality (BT + VR) and balance training (BT) groups. (**A**) single-leg stance on foam with eyes open, (**B**) single-leg stance on firm surface with eyes closed, (**C**) semi-tandem stance with visual input manipulations through VR. Dots represent individual data points and bars show the mean and SD. *Significant within-group differences between pre and post (* *p* < 0.05, ** *p* < 0.01, *** *p* < 0.001); # significant group × time interaction.

Table 2 presents descriptive statistics and the results of the ANOVA for pre- and post-intervention data for all measures of balance performance. The mixed ANOVA revealed a significant main effect of time for the single-leg stance on foam with eyes open and the semi-tandem stance on foam with visual input manipulations by VR (see Table 2). Significant within-group differences were observed in both groups for the single-leg stance on foam with eyes open (BT: *p* = 0.027, *d* = -0.54; BT + VR: *p* < 0.001, *d* = -0.53; Fig. 3A) and the semi-tandem stance on foam with visual input manipulations by VR (BT: *p* = 0.009, *d* = -0.91; BT + VR: *p* < 0.001, *d* = -1.25; Fig. 3C). A significant time × group interaction effect in favour of the BT + VR group was found for the single leg stance on firm surface with eyes closed (see Table 2). Post-hoc tests indicated a significantly decreased COP mean sway speed only for the BT + VR group (*p* < 0.001, *d* = -0.58; Fig. 3B). A tendency towards a significant time × group interaction effect was observed in the single-leg stance on foam with eyes open and in the single-leg stance on firm surface with eyes open (see Table 2).

### Model-based parameter identification

**Fig. 4:**
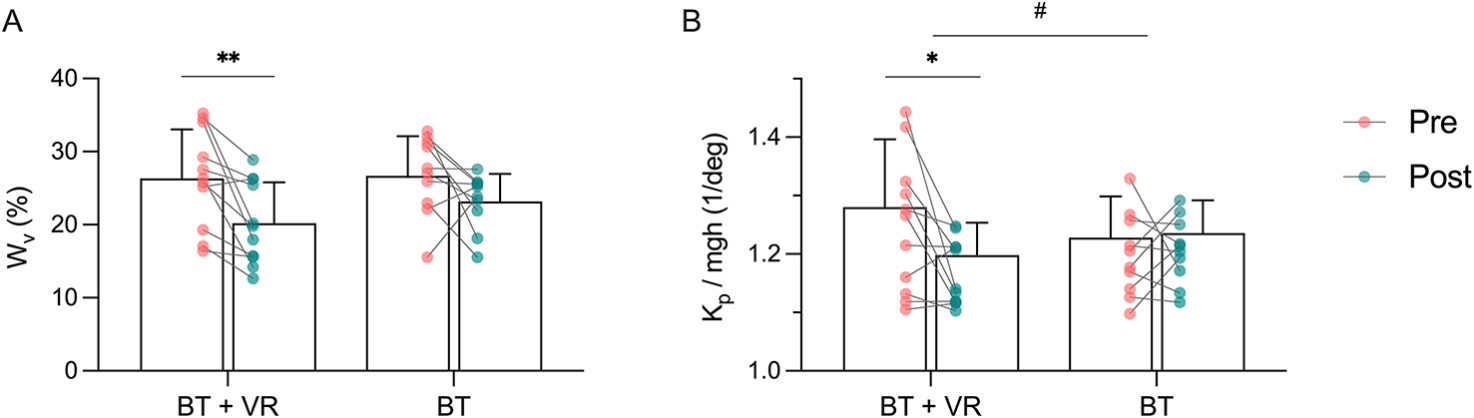
Balance control model parameters from pre- (red) to post- (green) exercise in the balance training with virtual reality (BT + VR) and balance training (BT) groups. (**A**) Visual sensory weight, (**B**) proportional feedback loop-gain, normalized by mgh (subject mass*gravitational constant*center of mass height). Dots represent individual data points and bars show the mean and SD. *Significant within-group differences between pre and post (* *p* < 0.05, ** *p* < 0.01); # significant group × time interaction.

Table 2 presents descriptive statistics and the results of the ANOVA for pre- and post-intervention data for the model-based parameter identification. The mixed ANOVA revealed a significant main effect of time for the visual sensory weight parameter W_v_ and the derivative feedback loop-gain K_d_ (see Table 2). Significant within-group differences in W_v_ were only observed for the BT + VR group (*p* = 0.007, *d* = -0.92; Fig. 4A). For the proportional feedback loop-gain K_p_, a significant time × group interaction effect was observed (see Table 2). Post-hoc tests indicated that the BT + VR group significantly reduced K_p_ following the training (*p* = 0.019, *d* = -0.82; Fig. 4B). For the derivative feedback loop-gain K_d_, post-hoc tests did not reveal significant within-group changes (BT: *p* = 0.051, BT + VR: *p* = 0.294).

## Discussion

This study sought to investigate the effects of BT versus BT + VR on balance performance and sensory integration in healthy young adults. By directly comparing these approaches, this study shed light on the potential and application of VR scenarios to modify the sensory integration mechanism underlying balance control and to enhance balance performance. The intervention in the BT + VR group involved tilting the visual scenery to induce conflicting sensory information. We hypothesized that this sensory conflict would lead to a downregulation of the unreliable visual sensory input, resulting in different adaptations in the sensory integration mechanism for balance control. Additionally, we expected that participants in the BT + VR group would demonstrate greater reductions in spontaneous sway compared to the group performing traditional balance training without VR^28^. Irrespective of group assignment, after the 4 weeks of balance training both groups showed an altered sensory integration mechanism underlying balance control. Participants relied less on visual information for balance control post-intervention compared to pre-intervention measurements. The BT + VR group showed improved balance performance in a balance task on firm ground and with eyes closed, despite not specifically training for this task. Conversely, the BT group, which trained with reliable visual input, did not exhibit these adaptations. Additionally, after the balance training the BT + VR group showed alterations in the sensory-to-motor transformation mechanism that converts sensory-detected body motion to corrective torque with significant reductions in the proportional and the derivative feedback loop-gain. The BT showed reductions only in the derivative feedback loop-gain. These findings support our initial hypotheses and underscore the versatility of adaptations elicited by VR-augmented balance training.

To build on the understanding of how balance training with and without VR affects sensory integration, it is important to consider the specific exercises and conditions involved in the training programs. The balance training program applied consisted of single-leg and semi-tandem stances on foam. During these exercises, the BT + VR group received visual input manipulations, whereas the BT group always had reliable visual input (eyes open). Participants in the BT group improved in the trained task, i.e., single-leg stance on foam, but showed no significant improvement in balance performance when the balance task was transferred to firm ground, irrespective of task complexity (i.e., eyes opened, closed). This finding is in line with the current literature and the specificity hypothesis, which consistently points to the task-specific nature of balance training effects^31,37^. While improvements in balance performance are notable in the trained task, they frequently fail to transfer to untrained tasks, even though tasks are similar. This phenomenon extends to the neural level, with task-specific adaptations in spinal and cortical excitability following balance training^38–40^. This specificity of adaptation hampers the real-world applicability of traditional balance training methods, particularly in varied environments and unpredictable settings that differ from the training conditions.

The BT + VR group, in contrast, also improved balance performance in single-leg stance on firm ground with eyes closed. Despite a different surface and a different visual condition compared to the trained task, the participants bypassed task-specificity of balance training and transferred balance gains to this untrained task. From a mechanistic point of view, this suggests that visual dependence for balance control decreased for participants in the BT + VR group at a behavioural level. This aligns with the findings from Lee at al^28^, who conducted a longitudinal study comparing balance training with visual disruption (using shutter glasses) and balance training with consistent visual input, examining their effects in balance performance and visual reliance. The authors found that balance training with visual disruptions led to greater balance improvements and reduced reliance on visual information to control balance. Additionally, previous studies observed improved balance control during quiet bipedal stance with eyes closed following repeated exposure to manipulated visual information in VR^29,41^. In contrast, improvements in balance control were not observed in quiet bipedal stance with eyes open, indicating that balance control in this visual condition remained unaffected after visual manipulations in VR. The authors interpreted this in terms of the sensory reweighting hypothesis and suggested a reduced reliance on visual sensory information for balance control. However, assessing balance on a behavioural basis (sway measures) has methodological constraints because major components of human balance control rely on closed loop feedback, where the control dynamics, including sensory integration, cannot be separated from internal noise sources in pure observational data, i.e., spontaneous sway measures^15,21^. The recording of COP/COM displacements encompasses all components involved, thereby offering an easy-to-administer marker to assess overall balance capacity. However, it cannot provide sufficient information to evaluate the subcomponents of the balance control system. To account for this limitation, the balance control system can be modelled as a closed-loop feedback control system. In this system, balance is dynamically regulated by active control mechanisms that continuously estimate body position via a sensory integration mechanism and generate corrective joint moments via a sensory-to-motor transformation mechanism to stabilize the body in space^11^. To examine the cause-and-effect relationships between sensory integration and motor action in a closed-loop control system, it is imperative to induce systematic external balance perturbations to the human body. These perturbations allow for the application of system identification methods to isolate and quantify the sensory integration and sensory-to-motor components^11^. In this study, we used a VR implementation of moving visual scene perturbations together with model-based interpretation of the results^22^. Therefore, this study is the first to dissect the sensory integration and sensory-to-motor transformation mechanisms in the closed-loop balance control system following balance training in healthy adults. The model-based analysis of the visual sensory integration mechanism revealed that participants in the BT + VR group relied to 26.4% on visual information to control balance in the pre-measurement and reduced this reliance by 23–20.2% in the post-measurement. This reduced reliance on visual information might facilitate general balance improvements in tasks where visual information is unreliable, absent, or negligible regardless of whether the task has been trained or not. According to the “Independent-Channel” control model proposed by Peterka^15^, a sensory weight represents the relative contribution of a particular sensory system to overall balance control. A relative reduction in one sensory weight must be associated with an increase in the relative sensory contribution of the other sensory modalities^11^. For example, Kneis et al.^42^ observed this plasticity in sensory weights following balance training in patients with peripheral neuropathy. The balance training resulted in an up-weighted proprioceptive input for balance control. Consequently, in our study, the down-weighting of visual information inherently led to a reciprocal upweighting of proprioceptive and/or vestibular information, which may have contributed to the improved balance performance in other balance tasks. However, it is important to note that our setup does not allow to distinguish whether the proprioceptive and/or vestibular contribution increased, as these mechanisms cannot be separated without additional external perturbations on the respective sensory modalities. Despite the BT + VR group showed improved balance performance during an untrained task, i.e., single-leg stance on firm surface with their eyes closed, this improvement was not replicated in another untrained task that involved standing on one leg on firm ground with eyes open. This observation suggests that participants learned to effectively reweight sensory information in situations where visual cues are unreliable or absent during their balance training. To further explore whether the reduced visual reliance bypasses task-specificity of balance training, future studies should test balance tasks with eyes closed on various devices that were not part of the training.

Another well-documented adaptation after balance training is the reduced cortical involvement in balance control^40,43^. Spinal contributions are either reduced by presynaptic inhibition^44^ or remain unchanged^40,43^. Muscular activity, conversely, was not significantly reduced after training. Taube et al.^45^ therefore concluded that balance training may lead to a shift in movement control from cortical to more subcortical and cerebellar structures. These structures are primarily responsible for orchestrating sensory integration to adjust the motor program stabilizing balance to the current situation^46,47^. However, direct evidence for adaptive responses within these subcortical structures to balance training is missing due to the challenges associated with the direct assessment of these structures. Our methodological approach cannot validate the hypothesis of increased subcortical contribution for balance control after balance training. Nevertheless, it supports the assumption of altered subcortical involvement by demonstrating that balance training can induce sensory reweighting and therefore change the extent to which sensory information is integrated. The adaptations in the sensory integration mechanism observed in both intervention groups extend beyond previous findings of spinal and cortical adaptations following balance training and suggest a more comprehensive reorganization of the neural substrates underlying balance control.

In addition to the changes in the sensory integration mechanism, balance training also affected the sensory-to-motor transformation mechanism that converts sensory-detected body motion to corrective torque. The sensory-to-motor transformation mechanism is operationalized by the feedback loop-gain K_p_ and K_d_^22^. K_p_ determines the strength of muscle contraction relative to the deviation from the desired upright position, reflecting how aggressively the neural controller responds. This is commonly referred to as the stiffness component of the controller because it dictates how rigid the control response is. On the other hand, K_d_ represents the muscle contraction relative to the deviation from desired body sway velocity, thereby providing a damping effect that helps to minimize oscillations or excessive movements. Whereas both feedback loop-gains were reduced in the BT + VR group following training, the BT group showed reductions only in the K_d_ component. A lower K_p_ indicates that fewer corrections are necessary because the body consistently remains closer to the desired state. Similarly, a lower K_d_ indicates that the body sway velocity is naturally more controlled and stable, requiring less damping to maintain balance. Functionally, the reduced feedback loop-gain along with reduced body sway suggest that the balance system is inherently more stable following balance training, with less need for corrective control actions.

The potential of VR-augmented balance training to alter visual sensory integration might provide practical implications for populations with increased reliance on visual information to control balance. For example, older adults^48^, patients with mild traumatic brain injuries^49^, vestibulopathy^50^, Parkinson’s disease^51,52^, chronic ankle instability^53^, or after anterior cruciate ligament injuries^54,55^ exhibit increased reliance on visual sensory information for balance control compared to healthy controls. A potential avenue to facilitate sensory reweighting for shifting the sensory reliance away from vision is to incorporate visual input manipulations in training and rehabilitation. The unreliable visual sensory information is down-weighted by the CNS, and neural processing may shift towards somatosensory information.

Additionally, our results might provide practical implications for the design of balance training to improve balance performance. Despite three of the four balance tasks did not reach statistically significant time × group interactions regarding COP sway speed, there is a noticeable trend towards significant interaction effects with generally large effects sizes across all balance tasks ($\:{\eta\:}_{p}^{2}$ = 0.13–0.23; see Table 2). This suggests meaningful and practically relevant effects in favour of the BT + VR training. The observed data patterns are consistent with theoretical expectations and previous research^28^, providing further evidence that the effects reflect systematic differences between the groups over time and are not random. Additionally, the study has achieved high statistical power for all balance tests (1-β > 0.99), indicating that the null hypothesis is accurately rejected and supporting the robustness of our findings.

Although the results of the present study seem to be quite unequivocal, there are some limitations that should be acknowledged. First, the balance training lasted only four weeks, with two sessions per week, 15 min per session. Thus, it could be possible that the training duration was not long enough to develop skills that could transfer to other balance tasks or that neural mechanisms can adapt. Nevertheless, the training parameters are within the range of various other balance training studies (for review see^2^). A meta-analysis by Bakker et al.^56^ further concluded that young adults improve balance performance rapidly within 1–3 training sessions. Additional training sessions did not further increase balance performance gains, which suggests that our balance training was sufficient to lead to balance performance adaptations. Further, Giboin et al.^39^ showed that already one day after acquiring a new balance skill, the neural adaptations can be observed in this balance task. In addition, task-difficulty in terms of support surface or sensory input kept constant for the BT group. Although this might have limited the potential for further performance gains, this was a trade-off we opted for to ensure the integrity of the study design and isolate the effects of the VR intervention. Second, the BT + VR group trained onsite due to the necessity of VR headset infrastructure, while the BT group conducted their training at home. Despite this difference, the BT group reported 100% adherence to the training regimen. The effect sizes for balance improvements in the trained task were comparable between groups, with the BT group showing an effect size of *d* = -0.52 and the BT + VR group showing an effect size of *d* = -0.53. Consequently, the location of the balance training does not appear to influence the balance outcomes. Third, the observed main effect of time on visual sensory integration, in the absence of a passive control group, cannot exclude the possibility that the differences observed between pre- and post-measurement could be attributed to the repetition of the test rather than the balance training programs. However, Assländer et al.^22^ demonstrated that our virtual reality setup provides reliable estimates of the human sensory integration mechanism and feedback loop-gains underlying balance (ICCs 0.7–0.92). Their reliability analysis was based on a day-to-day comparison, which suggests that there should be no effects of repeating the test after 4 weeks. Lastly, it needs to be mentioned that our findings from healthy young adults may not be representative of findings in clinical populations or individuals from different aging groups.

In conclusion, this study demonstrate that young healthy adults reduce their reliance on visual sensory information following balance training, indicating changes in the sensory integration mechanism underlying balance control. Additionally, balance training augmented with visual perturbations induced by VR reduces the feedback loop-gain that converts sensory-detected body motion to corrective torque. This underscores the potential of VR-based balance training to induce broad-based improvements in balance performance and sensory integration in healthy adults. By effectively reducing the reliance on visual input, VR-based training promotes a more flexible use of sensory information, enhancing balance control across a variety of conditions. These findings not only contribute to our understanding of the adaptations associated with balance training but also highlight the potential of VR as a powerful tool for enhancing balance training outcomes beyond the limits of traditional methods.

## Electronic supplementary material

Below is the link to the electronic supplementary material.

Supplementary Material 1

## Data Availability

The data is available from the corresponding author upon reasonable request.
