# Supplementary material for: Effects of balance training with visual input manipulations on balance performance and sensory integration in healthy young adults: a randomized controlled trial
Source: Sci Rep. 2024 Nov 19;14:28589. doi: 10.1038/s41598-024-79736-x (PMC11577058; doi:10.1038/s41598-024-79736-x)
Supplement: Supplementary file 1 — Supplementary Material 1 [file 41598_2024_79736_MOESM1_ESM.docx]

#
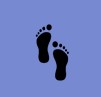
Exercise description

## Semi-tandem stance

Execution

- From the position with parallel feet, move one foot forward by half a foot length
- Do not overextend the legs
- Keep the toes stretched/no toe clawing
- Keep the eyes open

## Single-leg stance

Execution

- The knee of the standing leg should not be overextended
- The non-standing leg is slightly bent
- Keep the toes stretched/no toe clawing
- Keep the eyes open

**Session #1**

BT: eyes open

BT+VR. Visual scenery tilts of 10° @ 5°/s


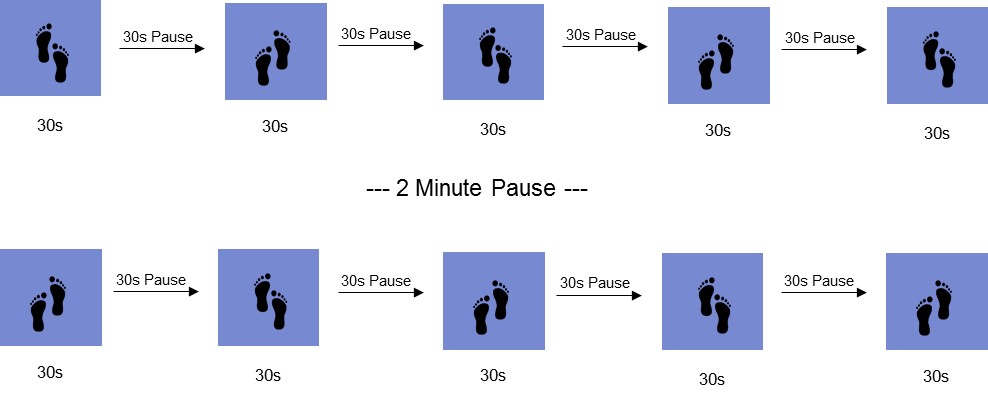


Comments:

**Session #2**

BT: eyes open

BT+VR: Visual scenery tilts of 20° @ 5°/s


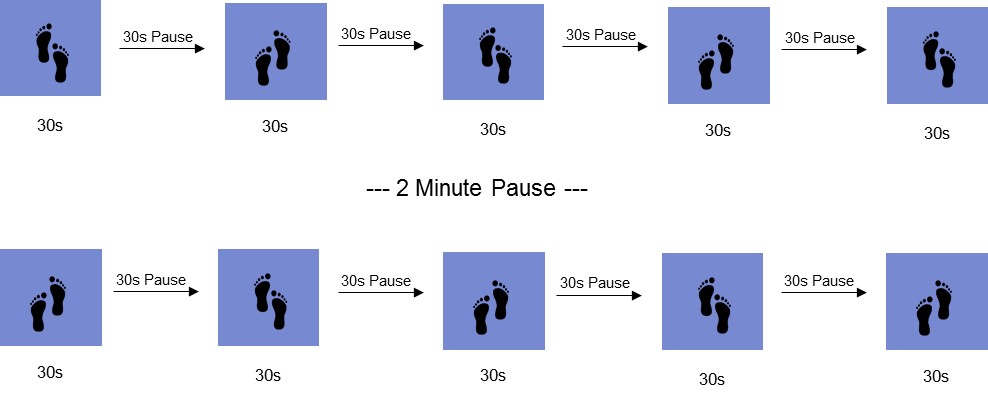


Comments:

**Training #3**

BT: eyes open

BT+VR: Semitandem: Visual scenery tilts of 20° ± 3° @ 5°/s | Single leg: 5° @ 5°/s


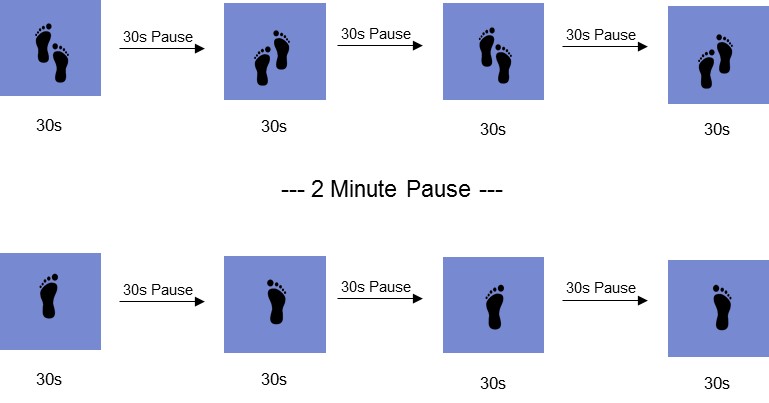


Comments:

**Training #4**

BT: eyes open

BT+VR: Semitandem: Visual scenery tilts of 20° ± 5° @ 5°/s | Single leg: 5° @ 5°/s


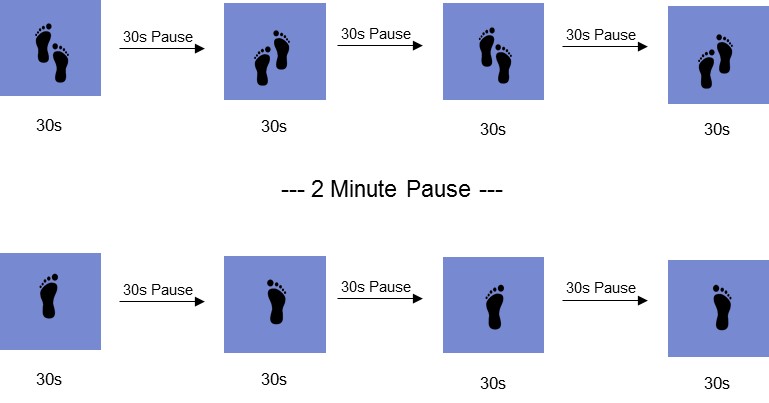


Comments:

**Training #5**

BT: eyes open

BT+VR: Visual scenery tilts of 10° @ 5°/s

**
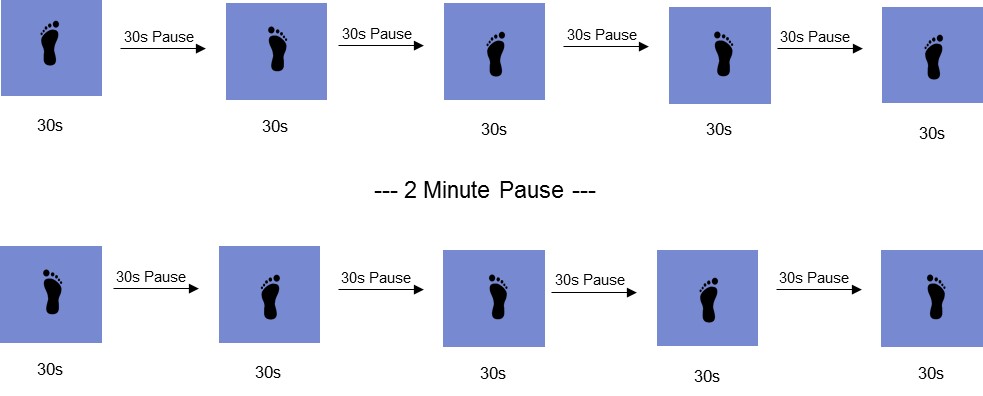
**

Comments:

**Training #6**

BT+VR: eyes open

BT+VR: Visual scenery tilts of 10° @ 5°/s

**
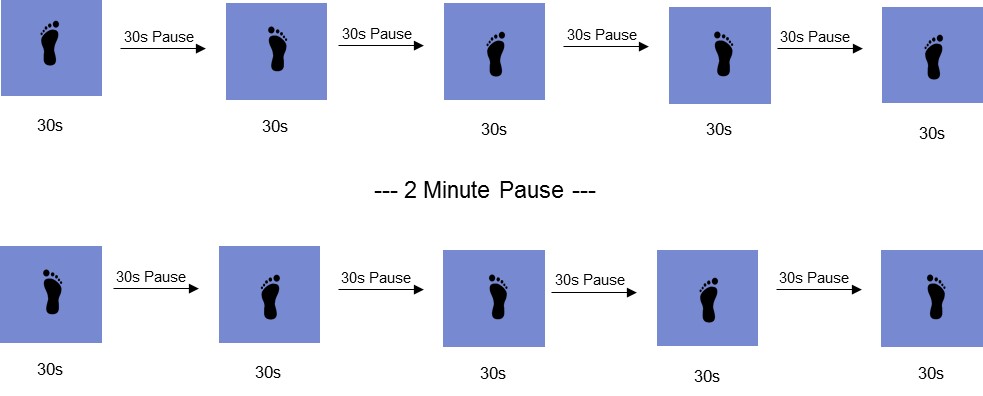
**

Comments:

**Training #7**

BT: eyes open

BT+VR: Visual scenery tilts of 10° ± 5° @ 5°/s

**
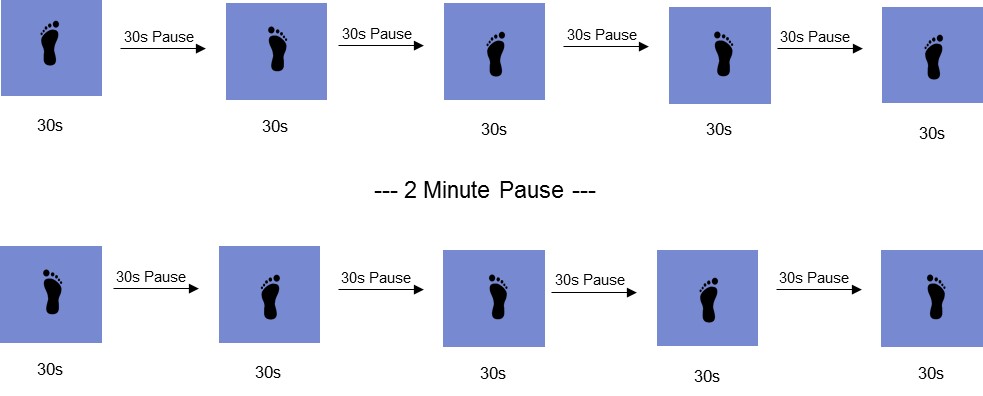
**

Comments:

**Training #8**

BT. Eyes open

BT+VR: Visual scenery tilts of 10° ± 5° @ 5°/s

**
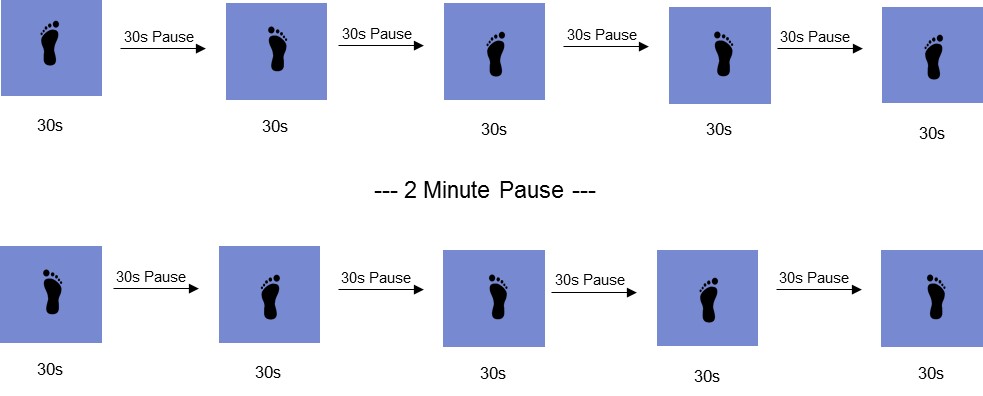
**

Comments:
